# Case Report: Histiocytic Necrotizing Lymphadenitis (Kikuchi–Fujimoto Disease) Concurrent With Aseptic Meningitis

**DOI:** 10.3389/fneur.2021.565387

**Published:** 2021-04-20

**Authors:** Yanna Song, Shan Liu, Lei Song, Huaqiu Chen, Miaoshui Bai, Jinhua Yan, Tianfei Luo, Kangding Liu, Li Sun, Yang Zhao

**Affiliations:** Department of Neurology and Neuroscience Center, The First Hospital of Jilin University, Changchun, China

**Keywords:** aseptic meningitis, Kikuchi-Fujimoto disease, histiocytic necrotizing lymphadenitis, case report, lymphadenopathy

## Abstract

Kikuchi–Fujimoto disease (KFD), also known as histiocytic necrotizing lymphadenitis, is a rare, benign, self-limiting disease characterized by local lymphadenopathy. Central nervous system involvement in KFD is extremely rare and remains a diagnostic challenge. Only 41 cases of aseptic meningitis associated with KFD have been reported worldwide, with just four cases (including our case) of KFD with meningitis as the first symptom. We report a case of KFD accompanied by aseptic meningitis with severely high intracranial pressure (400 mmH_2_O), increased white blood cell count (56 × 10^6^/L), and moderately elevated protein level (0.52 g/L). This case is unique in the delayed appearance of lymphadenopathy. After 1 month of treatment with steroids, fever, headache, and lymphadenopathy gradually disappeared, and the result of cerebrospinal fluid examination gradually became normal. In conclusion, based on our case findings and our literature review on KFD with aseptic meningitis, a diagnosis of KFD should be considered when delayed appearance of lymphadenopathy is observed in patients with aseptic meningitis.

## Introduction

Histiocytic necrotic lymphadenitis (HNL), first described by Japanese pathologists Kikuchi and Fujimoto et al. (1, 2) and also called Kikuchi–Fujimoto disease (KFD), is a rare local lymphadenopathy with a benign course and with clinical manifestations including fever, lymphadenopathy, rash, hepatosplenomegaly, central nervous system (CNS) symptoms, and hemophilic cell syndrome. KFD involves a variety of tissues and organs as well as the CNS, causing damage to the meninges, brain parenchyma, and peripheral nerves and even presenting neurological symptoms as prominent clinical manifestations or first symptoms. To date, most reports are based on the pathological features of KFD, and clinical reports of neurological damage as the first symptom are rare. Herein we discuss the case of a patient diagnosed with aseptic meningitis as the first clinical feature and who was ultimately diagnosed with KFD. Furthermore, we retrieved information on all patients diagnosed with KFD accompanied by aseptic meningitis from PubMed and conducted a comprehensive review of the etiology, clinical manifestations, auxiliary examination, diagnosis, prognosis, and treatment of this disease.

## Case Description

A 20-year-old Chinese man with a complaint of headache and fever for 5 days was admitted to our hospital. He had no specific medical history except for Ebstein anomaly corrective surgery at the age of 15 years. His family history was unremarkable. The temperature on admission was 38.1°C. Meningeal irritation signs, including neck stiffness and Kernig's sign, were absent. On admission, the laboratory test results revealed normal complete blood count, coagulation tests, erythrocyte sedimentation rate, procalcitonin, C-reactive protein, anti-streptolysin O, rheumatoid factor, tumor markers, anti-nuclear antibody (Ab), anti-dsDNA Ab, and anti-cardiolipin Ab. Serum antibodies against Epstein–Barr virus (EBV) showed a pattern of previous infection. Cranial computed tomography (CT) and CT venography were unremarkable. The initial intracranial pressure (ICP) measured was 220 mmH_2_O (normal range, 80–180 mmH_2_O) (Table 1, day 6 after symptom onset). Biochemical detection of cerebrospinal fluid (CSF) including protein, glucose, chlorine, and white blood cells showed that these were all within normal ranges. As meningitis was suspected, he was administered intravenously (IV) with acyclovir. However, the headache was not relieved after antiviral treatment. A second lumbar puncture (Table 1, day 11) revealed clear CSF with an opening pressure of 300 mmH_2_O. The CSF biochemical results were within the normal range. CSF cultures for bacteria, acid-fast bacilli, and fungi were also negative. Results of polymerase chain reaction (PCR) for herpes simplex virus, cytomegalovirus, human herpesvirus, EBV, and herpes zoster virus were normal. Meanwhile, he was administered IV with acyclovir, mannitol, and other symptomatic treatments.

**Table 1:** Cerebrospinal fluid examination.

**Days** **after symptom onset**	**Intracranial pressure** **(80~180 mmH**_**2**_**O)**	**Protein** **(0.15~0.45 g/L)**	**Glucose (2.3~ 4.1 mmol/L)**	**Chloride** **(119~129 mmol/L)**	**White blood cell** **count (0~8 × 10**^**6**^**/L)**	**Leukocyte classification** **(%)**
Day 6	220	0.36	3.7	120	6	Coenocyte 16 Monocytes 83
Day 11	300	0.32	3.7	128.7	3	-
Day 17	400	0.52	3.5	127.4	56	Coenocyte 8 Monocytes 92
Day 23	260	0.32	3.1	128.7	32	Coenocyte 99 Monocytes 1
Day 52	220	0.31	3.2	124.9	10	Coenocyte 10 Monocytes 90

**Figure 1:**
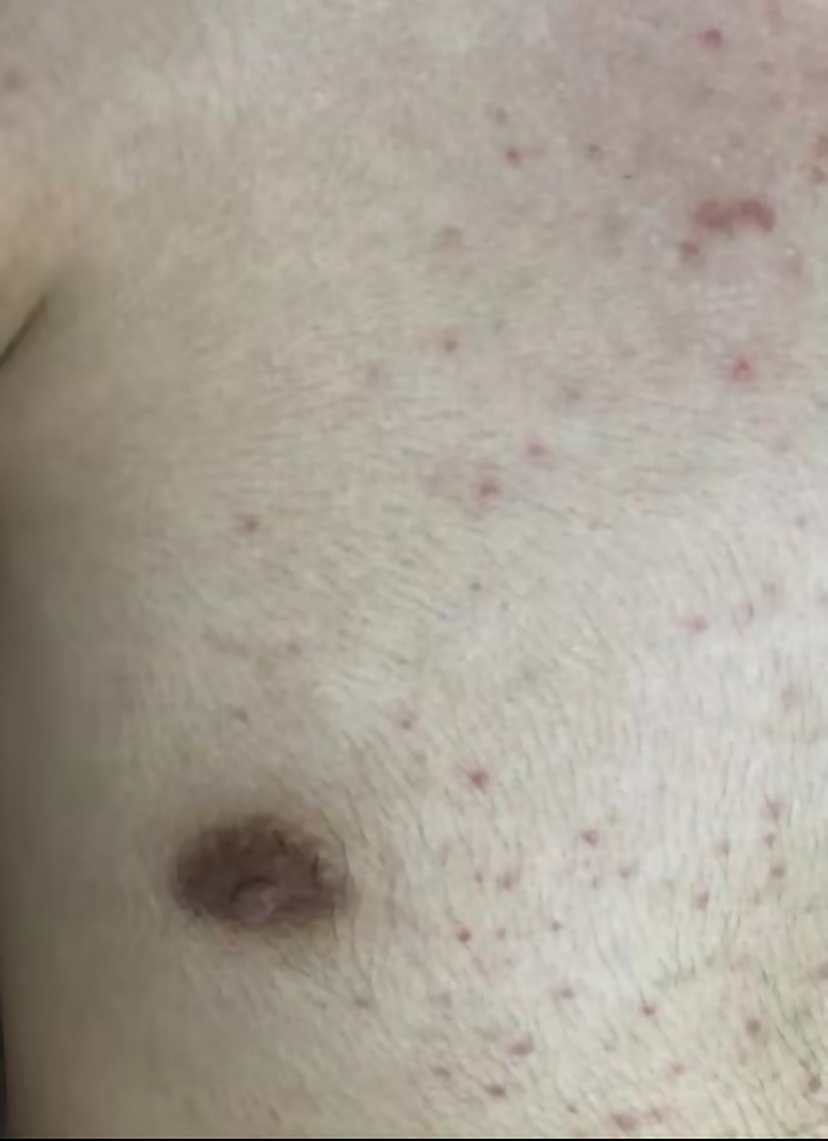
Erythema rash on the front of the chest.

On the 7th day after admission, the patient developed a severe headache accompanied by a rash all over the body (Figure 1). Neck stiffness and Kernig's sign were positive. Fever was essentially unaffected. Routine laboratory tests were normal as before. A third lumbar puncture examination (Table 1, day 17) showed a 400-mmH_2_O-high cranial pressure with elevated protein (0.52 g/L; normal range, 0.15–0.45 g/L) and white blood cell count (56 × 10^6^/L; normal range, 0–8 × 10^6^/L), among which monocytes represented about 92%, while glucose and chlorine levels were normal. The repeated CSF culture was sterile, and the PCR for viral agents was negative. At this point, mannitol was continued, and the dosage was increased to treat elevated ICP.

**Figure 2:**
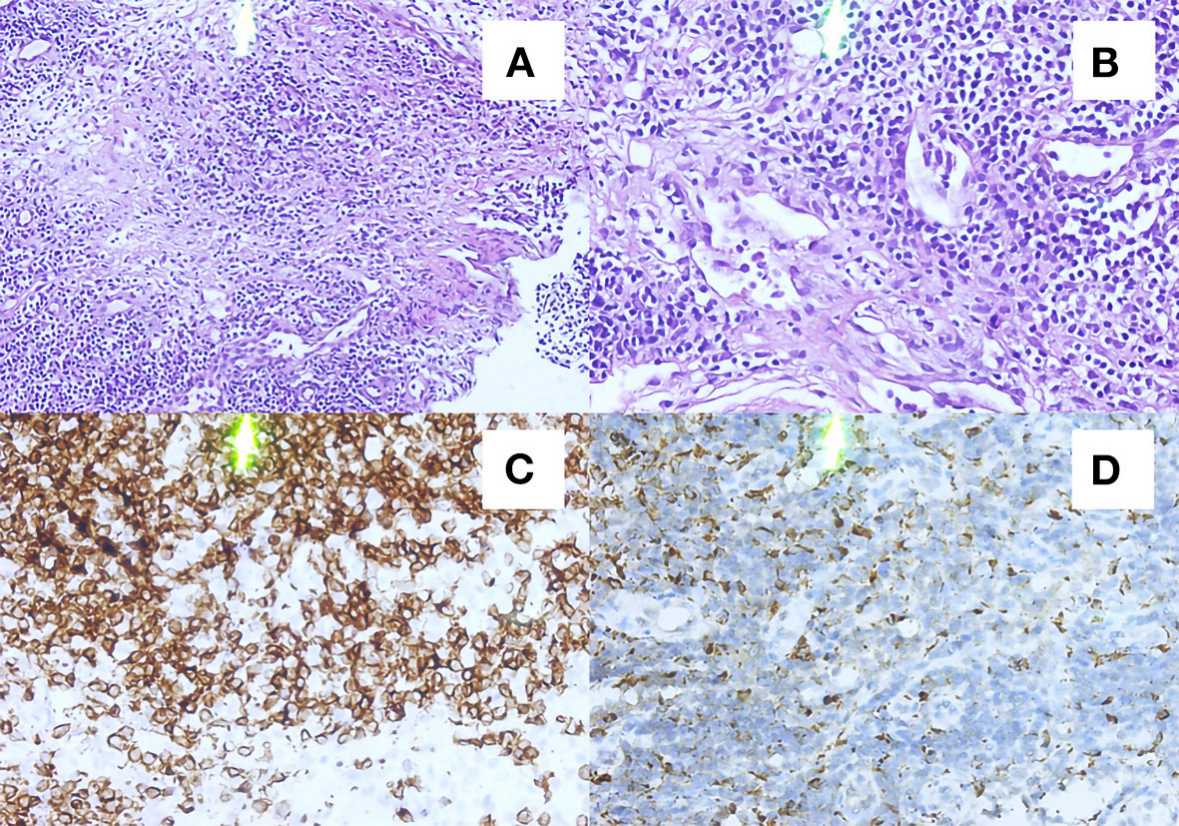
Histopathological and immunohistochemical findings of cervical lymph nodes. Hematoxylin–eosin staining showing hyperplasia, histiocytosis, and a few nuclear fragments in the left cervical lymph node sections. **(A,B)** Immunohistochemical staining showing CD3^+^ **(C)** and CD68^+^ **(D)** expression in the left side cervical lymph node sections.

On the 14th day after admission, several discrete, tender, and rubbery lymph nodes 10–20 mm in size were present on both sides at the posterior neck. Ultrasonography of the neck revealed multiple swollen bilateral cervical lymph nodes, some as large as 21 × 8.5 mm. A cervical lymph node biopsy demonstrated numerous lympho-histiocytic cells and karyorrhectic debris without neutrophils, suggesting KFD. Immunohistochemical staining showed CD68^+^ histocytes and CD3^+^ T cells, further indicating KFD (Figure 2). The absence of Reed–Sternberg cells, hematoxylin bodies, and neutrophils excluded the diagnosis of Hodgkin's disease and systemic lupus erythematosus (SLE). A fourth lumbar puncture (Table 1, day 23) revealed a decreased ICP of 260 mmH_2_O and WBC of 32 × 10^6^/L (monocytes 99%), while the other biochemical tests were normal. Based on the clinical manifestations and the CSF and cervical lymph node biopsy findings, the patient was diagnosed with KFD with aseptic meningitis. The treatment was adjusted to oral methylprednisolone administration (40 mg per day), which was tapered to 8 mg per day. During this period, the patient's clinical symptoms and CSF parameters (Table 1, day 52) gradually resolved. The patient was satisfied with the treatment outcome and had good compliance with follow-up. After 10 months of follow-up, no symptoms or signs of meningitis relapse or evolution into other autoimmune diseases were detected.

## Discussion

Herein we report an unusual case of KFD concurrent with aseptic meningitis. The patient presented with persistent fever, headache, skin rash, cervical lymphadenopathy, and positive meningeal irritation signs such as neck stiffness and Kernig's sign. The CSF culture was sterile, and lymph node biopsy revealed typical karyorrhectic debris and CD68^+^ histiocytes, which confirmed the diagnosis of KFD concurrent with aseptic meningitis. The patient's symptoms improved after treatment with steroids. In addition, the patient presented with less commonly reported symptoms, including initial aseptic meningitis and delayed lymphadenopathy, which further interfered with an early diagnosis of the disease. A literature review on the topic of KFD and aseptic meningitis was performed in PubMed using the following terms: “Kikuchi–Fujimoto disease aseptic meningitis,” “Kikuchi disease aseptic meningitis,” “histiocytic necrotizing lymphadenitis aseptic meningitis,” “Kikuchi–Fujimoto disease central nervous system,” “Kikuchi disease central nervous system,” and “histiocytic necrotizing lymphadenitis central nervous system.” We identified 41 cases (including the present case) to include in this review. Table 2 summarizes the epidemiological and clinical characteristics, laboratory features, brain MRI, prognosis, treatment, and outcomes of all cases.

**Table 2:** The clinical features of recurrent aseptic meningitis cases with Kikuchi–Fujimoto disease.

**Date/country**	**Gender/** **age**	Interval^*^ **(weeks)**	**CNS clinical manifestations**					**CNS duration (weeks)**	**CSF**				**Head CT or MRI abnormality**	**Steroids therapy**	**Outcome**	**References**
			**Headache**	**Confusion**	**Convulsions**	**Neck stiffness**	**Kernig's signs**		**Pressure (mmH_**2**_O)**	**WBC (×10^**6**^/L)**	**Protein (mg/dl)**	**Glucose (mg/dl)**				
2020/China	M/18	ND	+	+	–	–	–	1	300	454 ^MC^	400.1	Nor	+	+	C	(4)
2020/Kenya	F/29	#	+	–	–	+	+	3	ND	295^L^	56	Nor	–	+	R&C	(5)
2019/India	F/57	ND	+	–	–	–	–	3	ND	30 ^L^	80.47	66	+	+	C	(6)
2018/South Korea	M/12	3	+	–	+	+	+	2	ND	194^L^	312	52	+	+	C	(7)
2018/South Korea	M/17	2	+	–	–	–	–	1	ND	163^L^	333	D	+	+	C	(7)
2018/South Korea	F/15	1	–	+	–	–	–	ND	ND	87^L^	116	Nor	–	+	C	(7)
2018/India	F/20	0.5	–	+	–	+	–	4	ND	**I**^N^	299	69	ND	+	C	(8)
2018/China	M/20	#	+	–	–	+	+	4	400	56^MC^	56	63	–	+	C	Present case (2018)
2018/India	M/6	ND	+	–	–	–	–	12	ND	160 ^MC^	**I**	D	–	+	C	(9)
2017/Japan	M/19	ND	+	–	–	–	–	ND	ND	24^ND^	50	Nor	+	+	C	(10)
2017/America	F/19	1	+	–	–	–	–	ND	25.5	8^L^	Nor	Nor	ND	–	C	(11)
2016/India	F/15	2	–	+	–	+	+	4	ND	45^L^	28	76	+	+	C	(12)
2014/America	M/32	ND	+	–	–	+	+	ND	ND	14^L^	63	82	+	+	C	(13)
2014/America	M/9	3	–	+	–	+	+	2	>45	**I**^L^	77	ND	+	+	C	(14)
2013/China	F/25	ND	+	–	–	+	+	4	215	199^L^	126	46	–	+	R&C	(15)
2013/South Korea	M/28	ND	+	–	–	+	–	6	300	318^L^	285	60	–	+	C	(16)
2012/Japan	M/39	22 years	+	–	–	–	–	8	ND	33^L^	167	54	+	–	C	(17)
2012/Japan	M/28	ND	+	–	–	–	+	2	ND	27^MC^	31	**I**	ND	–	R&C	(18)
2011/Bangladesh	F/11	ND	+	+	–	–	–	2	ND	30^L^	89.7	42	–	+	C	(15)
2010/Japan	F/35	ND	+	–	–	+	+	ND	160	59^MC^	148	80	ND	+	R&C	(19)
2010/Turkey	F/36	7	+	–	+	–	–	12	ND	40^N^	61	49	+	+	C	(20)
2009/South Korea	F/13	4	+	–	–	–	–	ND	180	65^L^	28	56	ND	–	C	(21)
2008/Japan	M/29	ND	+	–	–	–	–	4	ND	63 ^ND^	ND	ND	ND	+	R&C	(22)
2007/China	M/25	#	+	–	–	–	–	4	260	15^ND^	128	48	–	+	C	(21)
2005/Britain	F/37	1/3	+	–	–	+	–	1	200	30^MC^	71	Nor	–	+	C	(23)
2005/South Korea	M/23	#	+	–	–	–	–	16	300	283^MC^	86	44	–	–	C	(24)
2003/India	F/17	ND	+	–	+	–	–	ND	ND	ND	**I**	ND	–	–	C	(25)
1999/Japan	M/27	6	+	–	–	–	–	5	200	78^L^	58	46	–	–	C	(26)
1999/Spain	M/14	3	+	+	–	–	–	12	ND	32^MC^	48	68	ND	+	C	(27)
1998/India	F/12	ND	+	–	–	–	+	ND	ND	100^ND^	ND	ND	–	+	R&C	(28)
1996/Japan	M/48	1	–	+	–	–	–	3	ND	135^L^	268	44	ND	–	C	(29)
1992/Japan	M/30	3	+	–	–	+	–	5	190	124^MC^	33	55	/	–	C	(26)
1990/Japan	F/21	3	+	–	–	–	–	3	150	109^MC^	42	61	/	–	C	(26)
1990/Japan	F/8	3	+	–	–	+	+	2	ND	49^L^	26	46	/	–	C	(26)
1989/Japan	M/13	3	+	–	–	+	+	4	ND	1685^MC^	198	59	/	–	C	(26)
1987/Japan	M/21	3	+	–	–	–	–	5	300	179^L^	200	65	/	–	C	(30)
1986/Japan	M/23	3	+	–	–	–	–	2	170	108^MC^	75	48	/	–	C	(31)
1985/Japan	F/16	2	+	–	–	+	+	2	180	95^MC^	51	46	/	–	C	(26)
1983/Japan	M/28	2	+	–	–	–	+	3	180	260^MC^	98	55	/	–	C	(26)
1979/Japan	F/38	6	+	–	–	–	–	6	150	89^MC^	42	39	/	–	C	(26)
1979/Japan	M/25	2	+	–	–	+	+	4	190	61^MC^	54	46	/	–	C	(26)

*CT, computed tomography; MRI, magnetic resonance imaging; CNS, central nervous system; M, male; F, female; C, cure; S, sequela; R, relapse; D, decrease; I, increase; Nor, normal; R&C, cure after relapse; MC, mononuclear cells dominant; L, lymphocytes dominant; N, neutrophile granulocyte; ND, not described*.

* *, interval between enlarged lymph nodes and CNS symptoms*;

*#, meningitis is the first symptom; –, absent; +, present; /, not available*.

HNL/KFD is a rare form of lymphadenitis of unclear etiology. Most scholars believe that the pathogenesis of KFD is related to viral infection and the auto-immune response is mediated by viral infection (3). In statistical terms, KFD is more common in young Asian women, with a peak age of onset between 25 and 29 years and a male-to-female ratio of 1:3–1:4 (3). In demographic terms, the distribution of KFD with meningitis has its own particular characteristics. Among the 41 cases of KFD with aseptic meningitis identified worldwide (Table 2), the ethnic origins of the patients were European (5%), North American (7%), African (2%), and Asian (86%). Japan had the highest prevalence worldwide, accounting for 41% of cases. The average patient age was 22 years (80% of the patients were <30 years old), and the sex (male/female) ratio was 1.28:1.

Commonly, fever and regional lymphadenopathy are the main clinical manifestations of KFD. In addition, KFD can cause uveitis, subretinal macular infiltration, acute renal failure, hemophagocytosis syndrome, interstitial pulmonary disease, and other rare complications (32). About 60–90% of patients present with long-term fever of unknown cause and temperature fluctuations ranging from 38 to 41°C. Posterior cervical lymphadenopathy is often the initial symptom, which is usually unilateral, accounting for 88.5% of cases (3). Up to 40% of patients with KFD may present with non-specific rashes, which may appear on the scalp, face, chest, back, and limbs, presenting with urticaria, rubella, erythema multiforme, papules, and papular abscesses (33). Our patient had a persistent fever after admission, with the highest temperature being 38.1°C. Several days after admission, the patient developed a skin rash and cervical lymphadenopathy, both of which suggested KFD. On the other hand, our patient developed delayed lymphadenopathy, an uncommon finding in KFD that complicates the diagnosis of KFD.

The clinical manifestations of KFD involving the CNS are complex and diverse, including meningitis, encephalitis, subdural effusion, optic neuritis, cerebellar ataxia, hemiplegia, and other signs (10, 23, 34–38). KFD concomitant with neurological symptoms is rare and prone to missed diagnosis and misdiagnosis. Aseptic meningitis is the most common CNS complication of KFD, accounting for 2.8–9.8% of KFD patients (39), mainly manifesting as headache, vomiting, and convulsion. Meningitis usually occurs 2–3 weeks after lymphadenopathy, while meningitis as a first symptom of KFD is rare (26). The course of KFD with aseptic meningitis usually takes 2–3 weeks, but a duration of 2–4 months has also been reported (26). A meta-analysis of 244 patients with KFD showed that 4.5% (11 cases) were associated with neurological impairment, including aseptic meningitis (eight cases), polyneuritis, or acute cerebellar ataxia (40). To date, of the 41 KFD patients with aseptic meningitis reported globally (Table 2), 88% suffered headaches, 19% had consciousness disorders, 7% had convulsions, 43% had neck stiffness, and 37% had positive Kernig's sign. As can be observed from Table 2, the average duration of CNS symptoms is 2–4 weeks, the longest is 4 months, and the shortest is 1 week. Among 41 patients with KFD with aseptic meningitis, 39 developed meningitis during the course of established KFD, while meningitis preceded other manifestations of KFD in only four patients. Moreover, the interval between lymphadenopathy onset and CNS symptoms was usually 1–3 weeks.

There is a lack of specific laboratory and imaging examinations for KFD diagnosis. CSF examination in patients with KFD concurrent with aseptic meningitis is sterile, with normal or slightly raised ICP, CSF protein, and leukocytes (26). In the literature review (Table 2), nine patients showed increased CSF pressure (200–400 mm H_2_O), and the CSF pressure in one case was only 25.5 mmH_2_O. Only nine KFD patients with aseptic meningitis had increased ICP, which may be related to the vasogenic cerebral edema caused by the increased permeability of cerebral capillary endothelial cells. CSF leukocytes were elevated (in most patients, these ranged from 8 to 300 × 10^6^/L, and one patient had 1,685 × 10^6^/L CSF leukocytes), with predominant mononuclear lymphocytes (86%). Furthermore, CSF protein was elevated (50–400 mg/dl) in 75% of patients, and the CSF glucose and chloride levels were mostly within the normal range. In patients with meningitis, irregular meningeal gadolinium enhancement and subdural effusion can be observed in enhanced magnetic resonance imaging (7). Among the 41 cases of KFD concurrent with aseptic meningitis (Table 2), 27% had imaging abnormalities. Although laboratory and imaging results have little significance as diagnostic criteria for KFD, they can still be used for differential diagnosis to exclude other diseases.

The definitive diagnosis mainly depends on excisional biopsy and histopathological and immunohistochemical examination. Therefore, in the absence of lymph node biopsy, KFD is easily misdiagnosed as tuberculosis, lymphoma, or connective tissue disease. The pathological changes associated with KFD are dynamic, and Kuo divided KFD into three types according to disease progression: proliferative, necrotic, and xanthomatoid (41). Lymph node biopsy showed pathologically disrupted lymphoid structures, histiocyte proliferation, and nuclear debris (42, 43). The typical immunohistochemistry findings of this disease are myeloperoxidase^+^ and CD68^+^ histiocytes (44, 45).

KFD is a benign, self-limiting disease, and most patients do not need special treatment (3). Moreover, it has a low recurrence rate (3 to 4%), and only several fatal cases have been reported (3, 5, 18). In the retrospective analysis of 11 cases (Table 2: cases 28 and 32–41) of KFD patients with meningitis, Sato et al. found that meningitis symptoms would spontaneously recede within 2–6 weeks without special treatment (26). As can be observed in Table 2, among the 41 cases of KFD with aseptic meningitis, 17 resolved spontaneously without steroid treatment, and six patients experienced a recurrence. Although there is no specific treatment for KFD, steroids may be used for patients with severe symptoms or with neurological lesions (23). Among all cases of KFD with aseptic meningitis (Table 2), 24 patients received steroid therapy, and 79% of them fully recovered.

Approximately 13–25% of KFD cases have been associated with SLE (40, 46). Many reports even refer to KFD as an early stage of SLE (3). However, the relationship between the two diseases remains unclear, and the concept of KFD being an atypical manifestation of SLE is still controversial (47). Considering this association, we recommend following KFD patients over time.

This case report has several limitations. On one hand, a definite diagnosis of KFD mainly depends on excisional biopsy, which is an invasive examination. On the other hand, due to the short follow-up time, we cannot predict whether the patient will relapse or develop SLE in the future. Therefore, long-term follow-up of patients with KFD is warranted. In addition, the pathogenesis of KFD-associated meningitis remains challenging for clinicians, thus deserving further study.

## Conclusions

Based on our observations, in patients with aseptic meningitis as the first symptom accompanied by cervical lymphadenopathy and rash, physicians should consider the diagnosis of KFD and perform lymph node biopsy at an early stage to avoid misdiagnosis and missed diagnosis and better guide the treatment. In addition, long-term follow-up should be performed in KFD patients with aseptic meningitis to monitor disease recurrence or progression to SLE.

## Data Availability Statement

The original contributions presented in the study are included in the article/supplementary material, further inquiries can be directed to the corresponding author/s.

## Ethics Statement

The studies involving human participants were reviewed and approved by the Ethics Committee of The First Hospital of Jilin University, China. The patients/participants provided their written informed consent to participate in this study. Written informed consent was obtained from the individual(s) for the publication of any potentially identifiable images or data included in this article.

## Author Contributions

YS conceived the idea, revised all the literature, and wrote the manuscript. SL and LSo collected the clinical data. HC, MB, JY, TL, KL, and LSu made tables and figures. YZ contributed to the revision of the manuscript and read and approved the submitted version. All authors have read and approved the final manuscript.

## Conflict of Interest

The authors declare that the research was conducted in the absence of any commercial or financial relationships that could be construed as a potential conflict of interest.

## Abbreviations

HNL: histiocytic necrotic lymphadenitis
KFD: Kikuchi–Fujimoto disease
EBV: Epstein–Barr virus
CSF: cerebrospinal fluid
SLE: systemic lupus erythematosus
PCR: polymerase chain reaction
CNS: central nervous system
Ab: antibody
CT: computed tomography
IV: intravenous
ICP: intracranial pressure.
